# Leisure time activities of Iranian patients with multiple sclerosis: a qualitative study

**DOI:** 10.15171/hpp.2016.08

**Published:** 2016-03-31

**Authors:** Seyed Mohammad Sadegh Hosseini, Ali Asgari, Mehdi Rassafiani, Farzaneh Yazdani, Mehrdokht Mazdeh

**Affiliations:** ^1^Department of Occupational Therapy, University of Social Welfare and Rehabilitation Sciences, Tehran, Iran; ^2^Department of Educational Psychology, Kharazmi University, Tehran, Iran; ^3^Department of Sport and Health Sciences, Faculty of Health and Life Sciences, Oxford Brookes University, Oxford, England; ^4^Department of Neurology, Hamedan University of Medical Sciences, Hamadan, Iran

**Keywords:** Leisure activities, Multiple sclerosis, Qualitative study

## Abstract

**Background:** Leisure time is one of the most important aspects of life, especially for people with chronic diseases. The concept and types of leisure have frequently been evaluated in different socio-cultural populations. The aim of this study was to identify the nature of leisure activities among a sample of Iranian patients with multiple sclerosis (MS) and classify the identified types of activities in the context of Iranian culture.

**Methods:** In this qualitative study, semi-structured interview was applied to gather data from 34 MS patients that were selected through purposive sampling. The interviews were continued up to the point of saturation. Content analysis was used to explore experiences of the interviewees regarding their leisure activities.

**Results: ** Six categories of leisure activities were extracted for the studied patients with MS i.e.physical, social, individual, art/cultural, educational and spiritual/religious.

**Conclusion: ** The results represented the range and heterogeneity of leisure activities amongst the MS patients. Considering participation in spiritual/religious and social activities as leisure time undertaking might reflect cultural diversity in the perception and use of time for recreation. For mental health promotion purposes, paying special attention to the types of activities that people of different socio-cultural background choose for their refreshment could help health care providers in giving tailored advice for patients with MS and other chronic debilitating disease.

## Introduction

Health of an individual is influenced by activities that she/he do as daily routines.^1^ Theories of occupational therapy consider human beings as complex systems that have dynamic relations with the surrounding environment.^2^ These interactions are critical for a productive life and take place in three domains: daily activities, work, and leisure. One of the most important goals in the occupational therapy is to keep the balance between activities of these three domains.^3^ If for instance there is not enough participation in leisure activities, the so called balance will be perturbed, which in turn affect health of individual. Leisure time activities are defined as “non-obligatory actions that are internally motivated and performed during free times, that is, time not committed to mandatory activities such as work, self-care, or sleep.”^4^ Participation in leisure activities is directly associated with self awareness, and is a part of healthy lifestyle.^5^ Therefore, it is related with health promotion measures and considered as an important factor in maintaining integrity of human functioning.^6^

Leisure activities however; could be limited by different medical conditions and disorders. One of the medical conditions that restrict leisure time activities is a neurological disorders which is called multiple sclerosis (MS)^7^ with a prevalence of 54.51 and an incidence of 5.87 per 100 000 (female/male sex ratio: 3.34: 1) in Iran.^8^ People with MS may suffer from a variety of complications like depression, fatigue and also cognitive impairment that limit their all daily routines, including leisure activities^9^ and therefore; negatively affect the sufferers well-being and quality of life.^10^ These patients generally spend their energy in performing daily maintenance and basic rather than leisure activities. Leisure time activities in these patients are simply ignored due to their focus on therapeutic measures to alleviate the symptoms and disabilities.^11^ This is while; participating in leisure activities may help in decreasing these symptoms or disabilities^12^ and thus reduce the burden of illness on patients’ families and larger society. The current alarming escalation in the number of MS patients in Iran require further scrutiny about the disease’s precipitating factors and patients’ quality of life. The later could by improved through the leisure activities that are fitted to the patients’ requirements.

Classification of leisure activities indicated a heterogeneity across studies.^13-15^ Packer for instance; considered four dimensions for leisure activities: instrumental, socio-cultural, physical, and passive measures. The physical dimension in this classification refers to leisure activities demanding high physical capabilities, and passive dimension to those with less physical demand.^15^ In other studies three classifications for leisure activities have been suggested: achievement (e.g. sports, playing musical instrument, painting or dancing), social (e.g. conversation, visiting friends or talking with them on the phone) and time-out domain (e.g. listening to music or watching TV).^16^

Participation in leisure activities in women with chronic fatigue disorder have been indicated to be through three main categories of actions: physical (i.e. strength, fitness, flexibility, and motion sports), social (i.e. relation with others to indicate the sense of empathy) and passive (the least social participation to cope with stress).^14^ Findings of another study on healthy individuals also added educational/creative dimension to leisure activities, which represents engagement in learning and activities of personal interests that are inspired with creative and intellectual stimuli.^17^

In addition to the observed diversities in the classification of leisure time activities across studies, their categorization may also be influenced based on theoretical frameworks. The intended and reported leisure activities therefore; could depend on cultural and social contexts of the participants in studies. In the past decades, investigations about cultural differences in performing and reporting leisure activities indicated considerable inconsistencies regarding explanation of the concept and categorization of leisure activities in different cultures. For example, in the North Americans’ culture emphasis is on human rights, freedom, happiness, individualism and independence in choosing or performing leisure activities while; in Asian cultures engaging in groups, strong interdependence, hierarchy and group harmonies are more emphasized.^18,19^

Such diversity in leisure activities was also reported among several ethnic groups living in a single country. Culture can influence the type, aim and level of participation in leisure time undertakings. Investigations on ethnic groups in the United States for instance; have revealed heterogeneity in the interests and level of participations in leisure activities. Regarding the type of preferred leisure activities, Hispanic/Latino groups were suggested to favor participation in music and dancing events, while Asian-Americans reported to usually select activities like board games, tile games, traditional cooking and arts. African-American groups were also declared to have interest in participation in running or jogging, woodworking and quilting as their leisure activities.^20^

All these socio-cultural differences in performing or reporting leisure activities were represented in the number and inherent differences of measurement tools. For instance, Nottingham Leisure Questionnaire (NLQ) includes items like membership in clubs and pet care,^13^ while Leisure Participation Scale has activities like bingo, bowling, golfing and lawn bowling,^14^ which are not well known in other cultures such as Iranian culture. Tondnevis, in a research about leisure in healthy persons between 20 and 65 years old, has showed that Iranian people are more interested in watching television, reading books, going to parties, going to parks, resting and speaking together. But dimensions of leisure activities were not investigated in this study.^21^

To the best of our knowledge there is no study to investigate leisure domains in the Iranian culture nor a disease-targeted tool or questionnaire to measure the level of participation or categorize leisure activities through the MS patients’ point of views.

Two questions were tried to be answered in this study: 1. What are the leisure activities of people with MS? 2. How the identified types of leisure activities can be classified? Main aim of this qualitative study was to identify corresponding items of leisure activities amongst the Iranian patients with MS and their appropriate classifications within the context of Iranian culture. We borrowed the holistic overview from the field of occupational therapy^4^ to clarify specially hidden aspects of the concepts that were used in different socio-cultural and temporal context^22,23^ by the Iranian MS patients.

## Materials and Methods

In this qualitative study, semi-structured face-to-face interview was applied to extract data and conventional content analysis was done for their analysis. In the content analysis, the concepts of leisure were scrutinized. The study data were examined based on the following steps: unit of analysis, meaning unit, condensing, abstracting, content area, code, category and theme as mentioned in Results.

Participants were 34 selected MS patients through the purposive sampling with diverse age, gender, work, education, and marital status structures. Inclusion criteria were being diagnosed as MS patient, ability to speak clearly for the data collection and personal interest to participate in the interview. Saturation was reached with 30 participants but we continued interview with 4 additional patients in order to provide greater confidence in the reliability of the study’s findings. Each participant was interviewed once in a general clinic that lasted for 20 to 60 minutes, with mean of 47 minutes. At the beginning of the interview session, the interviewer explained the purpose of the study to the participants and study questions were asked consequently. These questions were classified in two series. First series of questions were generally demographic questions to create rapport between the interviewer and interviewees. The second series of questions included: “How do you spend your free times?”, “What are your leisure activities on different seasons?”, “What activities do you do on holidays or vacations?” and “Does your disease (MS) has any impact on your leisure activities?”. When necessary, the interviewer used probes and clarifications. In addition to recording, field note has also been made in the data collection stage. The interviews were conducted in the same location, at the same time of the day, to keep the data collection conditions as consistent as possible. The interviews were performed from January to March of 2015.

The strategies that were used in this study to ensure the trustworthiness of our findings included triangulation, member check and, peer check. Triangulation performed through contrasting the interviews contents with field notes. Interpretations of the ten randomly selected participants’ explanations were sent to them for confirming and member checking. Peer check performed with eight experts who had an extensive experience in working with MS patients in order to review and confirm the extraction, coding, and categorization of data.

## Results

Participants of this study were thirty four people with MS (Mean age and SD: 38.85 ± 8.84), female/male ratio of the study respondents was 2.8:1, the lag time between the disease diagnosis and interviews’ date was from 1 to 30 years, educational range of the participants was from illiterate to academic, 82.4% of them were married and 38.2% were housewife. Details of demographic characteristics of the studied patients are shown in Table 1.

**Table 1:** Demographic information of MS patients

**Characteristics (n=34)**	
Age	
Mean±SD	38.85±8.84
Max	54
Min	23
Gender	
Female	25
Male	9
Marital Status	
Unmarried	5
Married	28
Divorced	1
Time of Diagnosis	
Mean±SD	7.47±6.30
Max	30
Min	1
Educational Level	
Illiterate	1
Less than high school diploma	9
High school diploma	13
Undergraduate	10
Postgraduate	1
Work	
Employed	24
Unemployed	7
Retired	2
Student	1

**Table 2:** An example of the steps of analysis

**Meaning unit**	**Condensation**	**Codes**
I have large gym ball, I have plan to exercise with it, 2-3 min every day.	Exercise with gym ball at home	Exercise at home
… a person should be beside me to help in doing stylish table decoration to welcome the guests.	Decorating table after coming guest	Table decorations
2 years ago Mecca and 4 to 5 years ago Syria, and 10 years ago Britain and Italy then I have gone when I was healthier than now.	Journey to Mecca, Syria, Britain, Italy	Travel abroad

**Table 3:** Overview of themes, categories, and subcategories with their frequency

**Theme**	**Category**	**Subcategory**	**Codes**
Leisure activities	Physical	Indoor	Exercise at home/Exercise on stationary bicycle/Dancing/…
		Outdoor	Walking/swimming/going to yoga class/…
	Social	Indoor	Going to parties/Talking with friends/relatives on telephone/Family gatherings/…
		Outdoor	Going to park/Going out with friends/Doing volunteer works/…
	Individual	Active	Playing game in cell phone /Gardening or growing flowers/Doing nonobligatory home activities/…
		Passive	Watching television/Listening to music/Reading books/…
	Spiritual/religious	Active	Participating in religious ceremonies/Attending in mosques for praying/Going to Quran classes/…
		Passive	Listening to religious dirge/Reading Quran or books of invocation /Reading spiritual stories/…
	Art-cultural	-	Knitting/Tailoring/Making cakes and pastries/…
	Educational	Teaching	Teaching children/Teaching English language/…
		Learning	Going to computer classes/Learning vitreous enamel/…

A qualitative content analysis was performed according to Graneheim and Lundman recommendations.^24^ Interviews were recorded by audiotape and transcribed verbatim. Every interview was considered as a unit of analysis. Content analysis started with reading the transcribed data obtained from the patients. Then phrases or parts of the text containing similar content were divided into meaning units. In the next step, the meaning units were condensed. An example of the process is presented in Table 2. Categories and subcategories were created from meaning units (See Table 3).

Table 3 shows main theme of the study which was leisure activities composing of six categories that have been retrieved from data analysis: physical, social, individual, art/cultural, educational and spiritual/religious.

### Physical category

Fourteen activities extracted for physical domain like going to yoga; swimming; doing exercise at home, walking, using a treadmill, dancing, and aerobic exercises; all of them demand a high physical activity. Physical category was divided into indoor and outdoor subcategories. Following two first sentences represented indoor physical activities, while two later sentences indicated outdoor physical activities:

“I have stationary bicycle and horizontal bar at home and do exercise with them some times”(P5).

“… In physical therapy clinic, he (practitioner) showed me to do some exercises at home and I practice them when I’m free”(P15).

“Since MS society arranged swimming pool designated times for us, I go to pool”(P29).

“I go to a yoga class with my friend”(P30).

### Social category

In this study the following definition of social activities has been used: activities which promote interpersonal interactions or social identity and regulate emotions. The social category with maximum frequency of activities included forty nine activities such as going to party, visiting friends, travelling inside the country, doing board games, talking with friends/relatives on phone, gathering together with family members and going to a park. Social activities were divided into indoor and outdoor subcategories as for physical category. Following sentences are representing the activities declared as examples for these subcategories. The first sentence is an example for indoor activity and the second for an outdoor one.

“Most of women like me talk with friends/relatives on telephone a lot and when I talk with them on phone, I don’t feel passing the time”(P2).

“… We [MS patients] gather in hospital on Thursday morning every week and discuss about our disease and problems …”(P6).

### Individual category

The third category, Individual activities, was defined as activities that allow the patients to do individually with personal stimuli. Our findings revealed 34 individual activities, such as: watching television, listening to music, working with computer, repairing house appliances, reading books and gardening. Two subcategories, active and passive activities were extracted from codes that formed this category. Active individual activities are those activities that require high physical demands but passive ones require less physical demands. The following sentences are the examples of active and passive individual activities, respectively.

“I have a large aquarium in my room and spend about one hour to clean it, feed the fishes and watch them”(P11).

“I’m at home most of the day; I usually spend my time with watching TV programs”(P3).

### Art/cultural category

These activities were defined as artistic activities that are culturally valuable for patients. The art/cultural category was comprised of 25 activities such as knitting; painting on pottery, tailoring and playing musical instrument. In this category, no subcategories were identified. Two example sentences indicating the reported type of activities in this group are mentioned below:

“My mom usually makes cookie on different occasions including for religious ceremonies or holy days and I help her”(P15).

“Sometimes, I craft leather accessories like bag and belt”(P2).

### Educational category

The educational category included several activities as engaging in learning or teaching along with attending educational classes based on personal interest and for intellectual promotion. Thirteen activities extracted from the codes and they were divided into two subcategories: learning and teaching activities. Participating in calligraphy, makeup courses and carpet weaving classes were examples of activities in learning subcategory while training knitting and teaching English language were indicative of activities in teaching subcategory. The types of activities in the subcategories were represented in sentences below:

“I usually go to … charity and participate in classes for learning painting and painting on pottery”(P24).

“Sometimes during school time, my niece comes to our house to do homework and I help her about 1-2 hours with her homework “(P29).

### Spiritual/Religious category

The last category was spiritual/religious activities that refer to behaviors that a person performs to connect or reinforce bonds with God. This class of activities contained activities like listening to religious songs, attending in mosques, and reading Quran and books of invocation. This category same as individual category was divided into active and passive subcategories. The following first sentence indicates an example of active and the next one a passive spiritual/religious activity.

“Some afternoons I go to mosque; this helps me to develop relation with others …”(P16).

“*Ziarate Ashura* [name of an invocation] is not performed as a routine, some people get relaxed because of the spiritual concepts”(P7).

## Discussion

Findings of the study revealed the studied MS patients’ leisure time activities which were based on their high to low frequency: social, individual, art/cultural, physical, educational and spiritual/ religious activities.

In this study activities of social nature was highly prevalent among the study respondents due to traditional importance of family structure in the Iranian culture and consequent family members and relatives’ support.^25^ In most of the Iranian families children live with parents and their siblings until they get married and by that time they receive emotional and economic support from their family members. Furthermore, helping family members and even strange people is encouraged in religious thought of Islam as the most practiced religion in the country.^26^ Such a valuable perspective is reflected in one of the *hadiths* of Prophet Mohammad (pbuh): “If you want God to help you, you help other people.”^27^ In the interviews some of the patients reported that they had been in contact with other patients to help them in managing their problems and coping with the illness. A considerable number of the married study participants also referred to the encouragements they had received from their partners or other family members for participation in several types of social activities. All these observations could be attributed to the regular cultural norms in the Iranian society.

Attending friend or familial parties was one of the common reported social activities by the study participants. However, some of respondents stated that they had not any interest to take part in such gatherings due to social and physical limitations. For instance, some of the patients stated that since they had not been able to play a kind of traditional ball game (*Vasatdari*) with others in parties that cause them to be embarrassed and lose their self confidence, they prefer not to attend a party in future. Social interactions is an important part of daily routines in the Iranian culture.^28^ Therefore; MS patients are expected like other ordinary people to spend a part of their times for socialization.

Doing social activities by the patients who suffer from debilitating chronic diseases were pointed out in several studies.^29-35^ Activities like doing unpaid volunteer work, going to cinema or a restaurant with friends or relatives, visiting friends or relatives at their homes, inviting friends or relatives who affected by degenerating disease such as Alzheimer disease to own home,^29^ talking with friends on phone; going out with friends, attending a party or an organized social event, participation in community wide political^33^ or family gatherings, visiting or talking with those neighbors who suffer from a chronic condition^14^ are the types of social activities that have been reported in the previous studies to be performed by the patients with disabling sicknesses. Most of these social activities were also proclaimed by the study participants that could be an indication for socio-cultural compatibility of the practiced activities across nations.

Based on the findings from a number of participants’ interviews, it was evident that some of the MS patients prefer to spend time alone. This might be explained by the respondents’ personality style or effects of the limiting disease on their mental health. Some of the patients stated that as a result of the consequent physical disability they had not been able to go out alone and without receiving assistance. Consequently, these people prefer to do individual self-paced activities at their homes. These patients also explained that they usually did not talk about their feelings to relatives and even their family members. Social labeling was the main reported reason to refrain from talking about the feelings of a patient who suffer from a severely disabling condition (being mad, depressed …).^36^ Classification of the reported activities were performed by the patients of disabling illnesses in other studies are almost identical with the classifications that were suggested in this study. Wang and Wang for instance; described motionless activities,^37^ Sered noted to the solitary category of activities^38^ and in a number of other studies the researchers explained passive category.^30,39,40^

Third category of the activities was objective and tangible, that were reported by many studied patients to maintain physical capabilities. Fear from lose of physical ability, reduction of fatigue and professional’s recommendations were the main reasons of the patients to do physical activities. One of the major concerns of the patients was fatigue that might influence other aspects of the patients’ life including decision about their leisure time.^41^ Eriksen and Bruusgaard study, revealed that physical leisure activities might decrease fatigue and therefore might improve patients’ health and well-being.^42^ Such a conclusion was also been made in other studies.^29,33,34,43,44^ Activities like walking for pleasure or excursion,^29^ aerobics, flexibility training, weight lifting, strength training or calisthenics, swimming, bicycling, and dancing^33^ are examples of the performed activities in these studies which are same with the reported activities of this category in this study.

The fourth category was art/cultural class of activities. Most of these activities however; were reported to be carried out by women patients especially those who were housewives because of their more available free times. The number of male participations in the study was much less than the female patients (19.3% versus 80.7%). Some of the activities of this category are relatively unique (carpet weaving) in some of the Iranian families and it might not be common in other countries. To the best of our knowledge only in the study of Yi et al doing art/cultural activities in leisure time was reported by the patients who survived a stroke.^45^ Performing creative activities in the Adolescence Leisure Interest Profile^46^ and doing craft works in Assessing Adult Leisure Activities^33^ however; were reported as art/cultural activities of the non-patient subgroups of the populations.

The fifth category was educational activities. Progressive nature of the disease increased physical disability as well as cognitive and emotional problems, but in this study, participants have indicated a tendency to have educational activities. On the other hand since, learning and teaching activities are generally time-consuming, frequency of these activities were less than the other above mentioned category of activities. Attending in art and craft classes was among the reported educational activities by Khemthong et al in the classification of leisure activities that were reported by the patients with non-chronic diseases.

The last category of activities was labeled as spiritual/religious type. The activities were reported by the subjects who had believed that these activities might have effects on their personal life. Activities like attending religious ceremonies might have different effect in different people. Some of the patients declared that they had interested in performing such activities while others had less interest to do so. One of the reasons to stay away from religious meetings by some of the patients was reported to be professionals’ recommendations regarding the negative psychological effects of these ceremonies on the patients’ mental health. The spiritual/religious type of activities in leisure time was not mentioned in other studies prevalently. However; “going to church” as an activity of leisure time was reported in two studies.^30,45^

Based on the findings of this research differences in leisure activities were observed between men and women. The male participants due to their familial financial role reported that they should pay more attention to their work and they have no time to devote in leisure activities.

A limitation of this study was the difficulty in recruiting accessing enough number of MS patients from both sexes. Because of the nature of MS disorder, generally the number of men with MS was 3.34 times less than women patient; therefore, balancing the number of male and female MS patients in this study was not possible. Further investigations are recommended to be done in future to provide empirical evidence about the potential differences might exist regarding the type of leisure activates between sexes. Application of a standard measurement scale in the quantitative studies may reduce measurement or recall bias.

## Conclusion

Findings of this study might provide baseline knowledge to prepare a culturally tailored measurement scale to assess the type and frequency of leisure time activities that potentially could be performed by MS patients. Results of such measurement will guide planners and health care providers in their recommended interventions to improve health of the sufferers and decrease the burden of the disease on individuals, families and societies.

## Ethical approval

Ethical approval was granted by the Ethics Review Board at the University of Social Welfare and Rehabilitation Sciences (USWR), Tehran. Informed consent was obtained from the study participants according to the recommended guidelines of the USWR before initiation of the interviews.

## Competing interests

The authors declare that there is no conflict of interest.

## Acknowledgments

We are grateful for all MS patients and specialists who participated in the study. This study is the first part of a PhD thesis entitled “Factor structure and developing a model for the leisure based on the Model of Human Occupation in the multiple sclerosis patients” in the University of Social Welfare and Rehabilitation. The author(s) received no financial support for the research, authorship, and/or publication of this article.
